# Are Bodybuilders at Higher Risk of Suffering Post-Tonsillectomy Haemorrhage?

**DOI:** 10.22038/ijorl.2018.32299.2064

**Published:** 2020-01

**Authors:** Petros V. Vlastarakos, John Plioutas, Christos Papastasinos

**Affiliations:** 1 *Department of * *Otorhinolaryngology* *, * *MITERA Infirmary, Athens, Greece.*

**Keywords:** Bodybuilding, Bleeding, Steroids, Supplements, Tonsillectomy

Elective tonsillectomy remains one of the most commonly performed operations in otolaryngology, with postoperative hemorrhage (PTH) being the most significant potential complication. Anecdotal evidence among ENT Surgeons suggested that bodybuilding athletes may be at increased risk of suffering from PTH, a notion corroborated by a recent case report, regarding primary PTH. The problem has been attributed to muscle-enhancing dietary supplements, or androgenic anabolic steroid abuse, albeit without satisfactory explanation regarding the mechanism of potential action. That is because, blood coagulation tests performed preoperatively are typically normal, and extensive blood coagulation workup postoperatively is not suggestive of any bleeding tendency.

We recently came across two cases of PTH in male patients practicing body building. Both were in their mid-twenties, had regularly been using muscle enhancing dietary supplements, and occasionally androgenic anabolic steroids; the first up to four months preoperatively, whereas the second was still an active user at the time of the tonsillectomy.

The first patient experienced five episodes of PTH, from the fourth until the 23^rd^ postoperative day, and required bipolar cautery in theatre on the first two occasions, with the latter three episodes being managed with oxygen peroxide-soaked tamponade in an outpatient setting. The second patient experienced persistent intraoperative bleeding and two postoperative PTHs on the first and 11^th^ day, requiring bipolar cautery in theatre. Pre- and postoperative blood coagulation workup were normal in both patients, and abdominal U/S did not suggest a fatty liver. Hence, the condition of the tonsillar vasculature remained as the last potentially implicated part of the patients’ bleeding tendency. Indeed, vascular fragility resulting from pressure and minor traumas is known to be a potential complication of hypercorticism (i.e. anabolic steroid abuse), and incipient vasculitis may be associated with high protein and amino-acid intake for muscle enhancement, due to the occurring oxidative stress and the ensuing free radical cascade.

PTH is a common emergency encountered in ENT surgical practice, affecting 2.5-4.1% of patients with normal coagulation studies. However, bodybuilding enthusiasts may represent a subgroup of an otherwise healthy population with higher potential risk of PTH, not only requiring appropriate preoperative informed consent, with frank account of the substances used for performance enhancement, but also increased perioperative vigilance, and prolonged period of postoperative observation. 

ENT Surgeons should be aware of the increased possibility of PTH even in apparently healthy and fit bodybuilding enthusiasts, and the necessity of repeated surgical management in most of these cases.

